# Disability weights for infectious diseases in four European countries: comparison between countries and across respondent characteristics

**DOI:** 10.1093/eurpub/ckx090

**Published:** 2017-09-11

**Authors:** Charline Maertens de Noordhout, Brecht Devleesschauwer, Joshua A Salomon, Heather Turner, Alessandro Cassini, Edoardo Colzani, Niko Speybroeck, Suzanne Polinder, Mirjam E Kretzschmar, Arie H Havelaar, Juanita A Haagsma

**Affiliations:** 1 Institute of Health and Society, Université catholique de Louvain (Clos Chapelle-aux-Champs, 30) Brussels, Belgium; 2 Department of Public Health and Surveillance, Scientific Institute of Public Health (WIV-ISP), Brussels, Belgium; 3 Department of Global Health and Population, Harvard School of Public Health, Boston, MA, USA; 4 Department of Statistics, University of Warwick, Coventry, UK; 5 European Centre for Disease Prevention and Control, Stockholm, Sweden; 6 Julius Center for Health Sciences and Primary Care, University Medical Center Utrecht, Utrecht, The Netherlands; 7 Department of Public Health, Erasmus MC University Medical Center, Rotterdam, The Netherlands; 8 National Institute for Public Health and the Environment, Centre for Infectious Disease Control, Bilthoven, The Netherlands; 9 Julius Center for Health Sciences and Primary Care, University Medical Center Utrecht, Utrecht, The Netherlands; 10 Department of Animal Health and Emerging Pathogens Institute, University of Florida, Gainesville, FL, USA; 11 Institute for Risk Assessment Sciences, Faculty of Veterinary Medicine, Utrecht University, Utrecht, The Netherlands; 12 Institute for Health Metrics and Evaluation, Seattle, WA, USA

## Abstract

**Background:**

In 2015, new disability weights (DWs) for infectious diseases were constructed based on data from four European countries. In this paper, we evaluated if country, age, sex, disease experience status, income and educational levels have an impact on these DWs.

**Methods:**

We analyzed paired comparison responses of the European DW study by participants’ characteristics with separate probit regression models. To evaluate the effect of participants’ characteristics, we performed correlation analyses between countries and within country by respondent characteristics and constructed seven probit regression models, including a null model and six models containing participants’ characteristics. We compared these seven models using Akaike Information Criterion (AIC).

**Results:**

According to AIC, the probit model including country as covariate was the best model. We found a lower correlation of the probit coefficients between countries and income levels (range *r*_s_: 0.97–0.99, *P* < 0.01) than between age groups (range *r*_s_: 0.98–0.99, *P* < 0.01), educational level (range *r*_s_: 0.98–0.99, *P* < 0.01), sex (*r*_s_ = 0.99, *P* < 0.01) and disease status (*r*_s_ = 0.99, *P* < 0.01). Within country the lowest correlations of the probit coefficients were between low and high income level (range *r*_s_ = 0.89–0.94, *P* < 0.01).

**Conclusions:**

We observed variations in health valuation across countries and within country between income levels. These observations should be further explored in a systematic way, also in non-European countries. We recommend future researches studying the effect of other characteristics of respondents on health assessment.

## Introduction

The Global Burden of Disease (GBD) studies, which started in the early 1990 s, presented an important set of comparative findings on the impact of different diseases, injuries and risk factors on population health.^1^^,^^2^ One of the most important results of these initiatives has been the introduction of the disability-adjusted life year (DALY), a new measure of overall disease burden, reflecting the number of healthy life years lost due to illness, disability or early death. This metric accounts for both premature mortality, expressed as years of life lost (YLLs) and time spent in states of reduced health, expressed as years lived with disability (YLDs). An essential parameter to calculate YLDs is the disability weight (DW), representing the severity of health loss associated with illness or disability. The DW is a value on a scale from zero to one: a DW of 0 means that a health state is equivalent to full health, whereas a DW equal to 1 means that a health state is equivalent to death. The value of a DW can be derived from the valuations of a panel of judges stated toward a set of hypothetical health states expressing the relative undesirability of the health state.^3^^,^^4^ There are three main types of health state valuation methods: (1) trade-off methods such as the time trade-off, and the person trade-off; (2) discrete choice experiments and (3) scales such as the Rating or Visual Analogue Scale or a combination of these.^5^

In 2012, an update of the GBD study was performed, including a new set of DWs for 220 unique health states.^6^ To assess the DWs, the GBD 2010 study proposed a new ordinal health state valuation methodology based on paired comparisons questions, a type of discrete choice question and a new population health equivalence technique, a type of trade-off method, to anchor DWs to the scale (from zero to one) aiming to address criticisms of previous approaches.^7–9^ With paired comparisons two descriptions of hypothetical health states are presented to respondents who have to decide which they regard as being healthier.

In 2008, the European Centre for Disease Prevention and Control (ECDC) launched an initiative to estimate the current and future burden of communicable disease in the European Union (EU) and European Economic Area (EEA) countries using DALYs.^10^^,^^11^

For the purpose of estimating the burden of communicable disease in Europe, the DWs developed for the GBD 2010 study^6^ had a number of limitations. The set of DWs was derived from a household survey based on responses from the general population of five non-European countries and from an open-access web-survey study performed in 167 countries with most of the participants from North America and Australia. This web-survey study was available in English, Spanish and Mandarin, whereas German (16%) is the most spoken language in Europe.^12^ Consequently the DWs were not based on the values representative of socio-demographic groups of the EU/EEA population.^13^ Moreover, DWs for 74 health states related to infectious diseases did not exist at all. Therefore ECDC, the Institute for Health Metrics and Evaluation and others started a collaboration to assess new DWs. They performed a web-based survey in Hungary, the Netherlands, Sweden and Italy^14^ because these four countries were considered geographically representative of the EU/EEA Member States.

The results of this study showed a high degree of agreement between the GBD 2010 and the European study. However, the putative differences in DWs between respondent characteristics with respect to their ranking of infectious diseases were not assessed. Previous studies already demonstrated that respondent characteristics such as age,^15^^,^^16^ income level,^17^ country,^18^^,^^19^ health status^20^ or profession^21^ may have an impact on health evaluation, and consequently on derived utility or DW. However, none of these previous studies specifically looked into the implications for DWs derived from pairwise comparisons. If significant differences in health valuation would exist in the derivation of infectious disease DWs, these may have to be taken into account in the selection of study populations of future studies aiming to assess infectious disease DWs or in the translation of study results to the general population.

The objective of the current study is therefore to compare DWs between the four European countries and across respondents’ characteristics.

## Methods

### Study design and participants

We performed a web-based survey between September and November 2013 in Hungary, the Netherlands, Sweden and Italy.^14^ The eligible study population was aged from 18 to 65 years. The participants were recruited through existing large panels of people available through the Growth From Knowledge (GFK) Company for participating in online surveys.

### Outcome trees

In addition to acute illnesses, infections can also lead to long-term consequences. To estimate the full burden caused by an infection, all health outcomes caused by the infection have to be estimated.^10^^,^^22^ This process, which relates all outcomes to their infectious causes results in an ‘outcome tree’ or a ‘disease model’,^23^ representing all health outcomes that are causally related to a hazard, their possible transition and transition probabilities in a flow chart.^24^ In total, 32 infectious diseases and six healthcare-associated infections were included in the burden of communicable disease in Europe study. These 32 infectious diseases and six healthcare-associated infections had 74 unique health states for which DWs were evaluated in this study.^24^

The short health state descriptions (35 words or fewer) were developed by Salomon et al. to emphasize the major functional consequences and symptoms associated with each health state with simple non-clinical vocabulary. The health state descriptions used in this study are available from Ref. 14.

### Procedures

We adapted the questionnaire developed by Salomon et al.^6^ by including additional questions about disease experience and by changing income and educational categories to fit the situation in the four countries. For disease experience status we asked if the participant suffered from a disease or the consequences of an injury. We used existing health state descriptions developed by Salomon et al.^6^ for 74 health states related to infectious diseases.

Three different versions of the questionnaire were designed. Each version included questions about demographics of the participant (age, sex, educational and income level and disease experience), and each participant received only one version of the questionnaire. The first version was composed of 15 pairwise comparison (PWC) questions on chronic (life-long health) states and three population equivalence questions (PHE). The second version of the questionnaire included 15 paired comparison questions on temporary (lasting 1 week) health states and three PHE questions. The third version included five PWC questions with a chronic framing to accommodate PHE questions questions and three PHE questions. PHE questions, which allow to anchor the final DWs from 0 to 1 and to elicit trade-offs between mortality and nonfatal health states, were finally not used in the European DW study. For the PWC questions, participants were asked to imagine that two people have the same number of years left to live, and that they will experience the health problems described for the rest of their lives (or for 1 week for temporary health state). Then, the respondents were presented with two descriptions of hypothetical people, each living in a particular health state, and then asked which person they regarded as being healthier.

For instance:

− ‘*The first person has diarrhea three or more times a day with occasional discomfort in the belly’*.
− *‘The second person has a low fever and mild discomfort, but no difficulty with daily activities.’*

*Who do you think is healthier overall, the first person or the second person?’*

The questionnaires and health states were translated from English to Hungarian, Dutch, Swedish and Italian and then checked by an independent native speaker.

The questionnaire was assigned to participants through a computer algorithm that first attributed the study version with the lowest percentage of participants at the time of assignment. Then after the version was allocated, the algorithm selected the health states to include in the questionnaire based on the minimum number of allocations that the health state had at that moment.

### Statistical analysis

To assess the representativeness of our study population, we applied chi-squared tests to assess the comparability of the age, sex and educational distribution of the study population with those of the respective national populations. Because income levels were categorized, we assigned the midpoint of each income category to all respondents in that category, which we compared with mean household income values from Eurostat.^25^

To investigate the differences between health states, we ran probit regression analyses on the choice responses. The first health state of the pair had a value of 1, the second health state of the pair had a value of –1 and those not in the pair had a value of 0. The resulting probit regression coefficients for each health state expressed the relative differences in valuation of the health state on an arbitrary scale. To project the results from the probit regression model on a DW scale ranging from zero to one, we ran a locally weighted scatterplot smoothing regression model (loess) of the probit regression coefficients vs. the logit transformed GBD2010 DWs.^14^ We then predicted logit transformed DWs for each of the probit coefficients from the loess fit. To change the scale and obtain values ranging between 0 and 1 we applied an inverse logistic transformation of these predicted DWs. We estimated uncertainty around the mean DWs through a bootstrap procedure. We first generated 200 samples of the pairwise coefficients using their probit estimated mean and standard deviation. We then used the generated samples to calculate 200 loess fits. We generated 200 samples for each of the DWs using loess fit, producing 40 000 bootstrap samples for each DW. We finally calculated uncertainty intervals (UI) as the 2.5th and 97.5th percentile of the corresponding distribution of bootstrapped samples intervals for the DW. These analyses were performed using the overall database of 255 health states available in Haagsma et al.^14^ We hereby extracted the 74 probit coefficients and DWs of the health states relating to infectious diseases, which were the primary focus of this study.

We performed the same analysis of the responses across countries, sex, age, disease experience status, income and education level and within countries by sex, age, disease experience status, income and education level.

The Spearman correlation coefficient was also used to evaluate the relation of both the probit coefficients between countries, age groups, sex, disease experience status, educational and income level.

To evaluate the effect of participants’ characteristics, we also estimated difference between two health states in models with interactions that allow each country, or each population subgroups to have its own set of distinct values, assuming that the difference between the two state values was normally distributed. We then compared models using Akaike's Information Criterion (AIC).

We interpreted a *P* value < 0.05 as significant. Analyses were done with R (version 3.0.2) and using the speedglm package.^26^

## Results

### Characteristics of respondents

Overall, 30 660 respondents completed the DW survey of which 6053 participants (20%) were from Hungary, 8054 (26%) from Italy, 8005 (26%) from the Netherlands and 8548 (28%) from Sweden.

The average age was 41.5 years (standard deviation [sd] 13.3) in Hungary, 41.7 years in Italy (sd 12.3), 44.1 years in the Netherlands (sd 12.9) and 41.9 years in Sweden (sd 13.7). Approximately half of the respondents were male in each of the four countries.

The majority of the respondents had a medium educational level in Hungary (49%), Italy (48%) and Sweden (51%) but while in the Netherlands the level of education of the participants was more equally distributed (low: 35%, medium: 35% and high: 30%).

Most of the respondents had a low income level in Italy (47%), medium income level in the Netherlands (56%) and Sweden (50%) and high income level in Hungary (47%) (table 1).

**Table 1:** Description of the study population

	Total	Hungary	Hungarian population	*P*-value^b^	Italy	Italian population	*P*-value^b^	Netherlands	Dutch population	*P*-value^b^	Sweden	Swedish population	*P*-value^b^
	(*n* = 30660)	(*n* = 6053)	(%)^a^		(*n* = 8054)	(%)^a^		(*n* = 8005)	(%)^a^		(*n* = 8548)	(%)^a^	
**Sex**													
Men	14 719	2889	47.6	0.999	3886	48.4	0.999	3842	49.5	0.943	4102	49.9	0.902
	(48.0%)	(47.7%)			(48.2%)			(48.0%)			(48.0%)		
**Age**				0.993			0.982			0.584			0.869
18–34 years	9574	2047	34.1		2522	30.2		2148	33.3		2857	36.5	
	(31.2%)	(33.8%)			(31.3%)			(26.8%)			(33.4%)		
35–49 years	10 753	2026	34		2983	38.1		2883	34.3		2861	33.4	
	(35.1%)	(33.5%)			(37.0%)			(36.0%)			(33.5%)		
50–65 years	10 333	1980	31.9		2549	31.7		2974	32.4		2830	30.1	
	(33.7%)	(32.7%)			(31.6%)			(37.2%)			(33.1%)		
**Educational level**				0.242			0.49			0.95			0.546
Low	9125	2344	80.9		2668	86.1		2801	71.4		1312	69.9	
	(29.8%)	(38.7%)			(33.1%)			(35.0%)			(15.3%)		
Medium	14 000	2976			3896			2804			4324		
	(45.7%)	(49.2%)			(48.4%)			(35.0%)			(50.6%)		
High	7535	733	19.1		1490	13.9		2400	28.6		2912	30.1	
	(24.6%)	(12.1%)			(18.5%)			(30.0%)			(34.1%)		
**Income level (*n* = 23950)**				na			na			na			na
Low	9458	1301			3177			2053			2927		
	(39.4%)	(30.0%)			(47.2%)			(35.3%)			(41.4%)		
Medium	10 879	1016			3083			3271			3509		
	(45.4%)	(23.4%)			(45.8%)			(56.3%)			(49.7%)		
High	3613	2026			472			484			631		
	(15.1%)	(46.6%)			(7.0%)			(8.3%)			(8.9%)		
Mean [SD] (€)		6778 [3276]	5469		25764 [17723]	18997		39 839 [23 850]	23 802		23 404 [12 541]	27 728	
**Disease experience**				na			na			na			na
Yes	8900	2085	NA		1448			2337(29.2%)	NA		3026	NA	
	(29.0%)	(34.4%)			(18.0%)								
											(35.4%)		

a According to Eurostat: http://ec.europa.eu/eurostat/data/database (2013 population).

b *P* values resulting from the chi-square test.

NA: Not Available.

na: not applicable.

Approximately one in three respondents reported that they were suffering of a disease or of the consequences of an injury in Hungary (34%), the Netherlands (29%) and Sweden (35%) and one in five respondents in Italy (18%) (table 1).

Comparison of the study population with data from Eurostat showed that the study population was representative of the national populations in the four countries in terms of sex distribution, age distribution and educational levels.^27^ For income levels we observed that, except in Sweden, mean income of the respondents was higher than in the general population table 1.

### Probit regression models

We observed that all models, except the model including disease experience status (model 3) (AIC = 417 722), had lower AIC than the model without covariate (AIC = 417 572). We also observed that the model including country as covariate had the lowest AIC (411 142), the next lowest being the based on age (AIC = 416 940), then on educational level (AIC = 417 178), on sex (AIC = 417 271) and finally on disease experience status (AIC = 417 722). That means that probit model including country as covariate was the best model because AIC represents the loss of information caused by using models to represent the process generating the actual data. We did not compare the AIC for the model including income level as covariate with the other models because it included less data because of the missing answers (table 2).

**Table 2:** AIC of seven probit models, including a null model and six models containing one covariate, i.e. country, sex, age, disease experience status, income level, or educational level

Probit models	AIC
(1) Null model	417 572
(2) Country	411 142
(3) Disease experience status	417 722
(4) Sex	417 271
(5) Educational level	417 178
(6) Income level	NA^a^
(7) Age	416 940

^a^Not available: AIC not calculated because it included less data than the other models.

### Probit regression coefficients–correlation coefficients

#### Between country

All correlation coefficients were significantly different from zero (*P* < 0.001).

**Figure 1:**
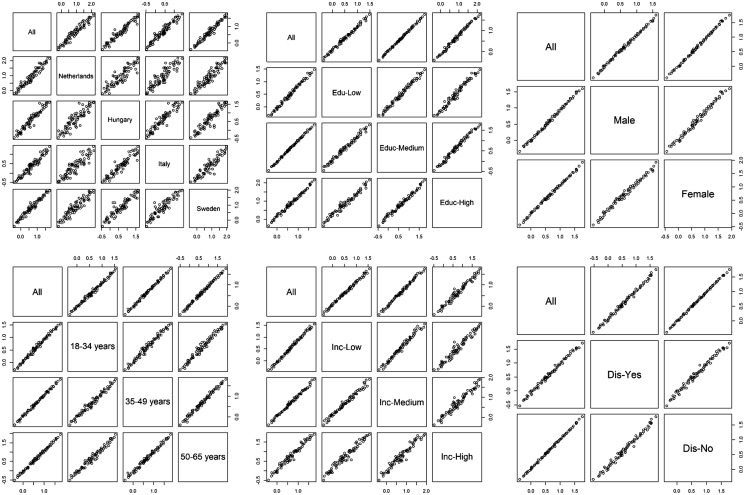
Correlation matrix of probit coefficients by country, educational level, sex, age, income level and disease experience status

Overall, we found a lower correlation of the probit coefficients between country than between age-groups, educational, income, sex and disease experience status (figure 1). The highest linear correlation of the probit coefficients was found between Italy and Hungary (*r*_s_ = 0.95) and the lowest correlation was observed between Hungary and the Netherlands (*r*_s_ = 0.89) (figure 1).

The correlation of the probit coefficients was high between age groups (range *r*_s:_ 0.98–0.99), educational levels (range *r*_s_: 0.98–0.99), sex (*r*_s_ = 0.99) and disease experience status (*r*_s_ = 0.99) and lower for income levels (range *r*_s_: 0.97–0.99).

#### Within-country

All correlation coefficients were significantly different from zero (*P* < 0.001).

We observed a higher correlation of the probit coefficients by age-groups in the Netherlands (range *r*_s_: 0.97–0.98) than in Hungary (range *r*_s_: 0.95–0.96), Italy (range *r*_s_: 0.95–0.97) or Sweden (range *r*_s_: 0.96–0.97). In all countries, the highest correlation was observed between 35–49 years and 50–65 years groups. In every country, except for the Netherlands, the lowest observed correlation was between 18–34 years and 50–64 years. In the Netherlands, the lowest observed correlation was between 18–34 years and 35–49 years groups (*r*_s_ = 0.97).

We observed roughly the same pattern for educational levels. The correlation of the coefficients was higher in the Netherlands (range *r*_s_: 0.97–0.98) than in Hungary (range *r*_s_: 0.94–0.98), Italy (range *r*_s_ ≥ 0.94–0.96) and Sweden (range *r*_s_: 0.95–0.98). In every country, except Sweden, the highest correlation was observed between low and medium educational level. In Sweden the highest correlation was observed between medium and high educational level (*r*_s_ = 0.98). Except for the Netherlands, the lowest observed correlation was between low and high educational level. In the Netherlands, the lowest observed correlation was between medium and high educational groups (*r*_s_ = 0.97).

We observed relatively less concordance of the responses between income levels across country. The correlation of the coefficients was better in Hungary (range *r*_s_: 0.94–0.95) than in the Netherlands (range *r*_s_: 0.92–0.98), Italy (range *r*_s_: 0.89–0.95) and Sweden (range *r*_s_: 0.93–0.98). The lowest correlation was observed between medium and high income levels in the Netherlands (*r*_s_ = 0.92) and between low and high income levels in Hungary (*r*_s_ = 0.94), Italy (*r*_s_ = 0.89) and Sweden (*r*_s_ = 0.93). The highest correlation was observed between low and medium income levels in the Netherlands (*r* = 0.98) and Sweden (*r* = 0.98) and between medium and high levels in Hungary (*r*_s_ = 0.94) and Italy (*r*_s_ = 0.93).

We observed a higher correlation of the coefficients between disease experience status in Sweden (*r*_s_ = 0.99) than in the Netherlands (*r*_s_ = 0.97), Hungary (*r*_s_ = 0.97) and Italy (*r*_s_ = 0.96).

In all countries, we observed a high correlation of the probit coefficients between sex (range *r*_s:_ 0.97–0.98).

Overall, within country, we observed a lower correlation of the coefficients by income levels than by age groups, educational levels, sex and disease experience status.

Except for income level, Dutch participants showed higher correlation of the probit coefficients for all participants’ characteristics.

### Disability weights

‘Distance vision, mild impairment’ had the lowest DW in Hungary [H]: 0.007 [95% UI 0.004–0.013], Italy [I]: 0.004 [95% UI 0.002–0.008] and Sweden [S]: 0.005 [95% UI 0.003–0.009]). ‘Hearing loss, mild’ had the lowest DW in Netherlands [N]: 0.004 [95% UI 0.002–0.009]. ‘Intensive care unit admission’ had the highest DW for all the countries (H: 0.597 [95% UI 0.464–0.690]; I: 0.530 [95% UI 0.430–0.617]; N: 0.624 [95% UI 0.493–0.719] and S: 0.717 [95% UI 0.552–0.766]) (table 3).

**Table 3:** Comparison of the rank order (descending) of the health states between countries

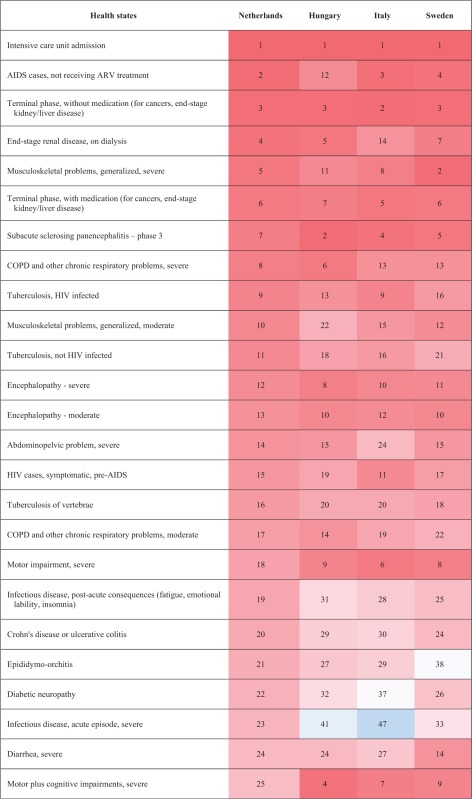
	(Continued)


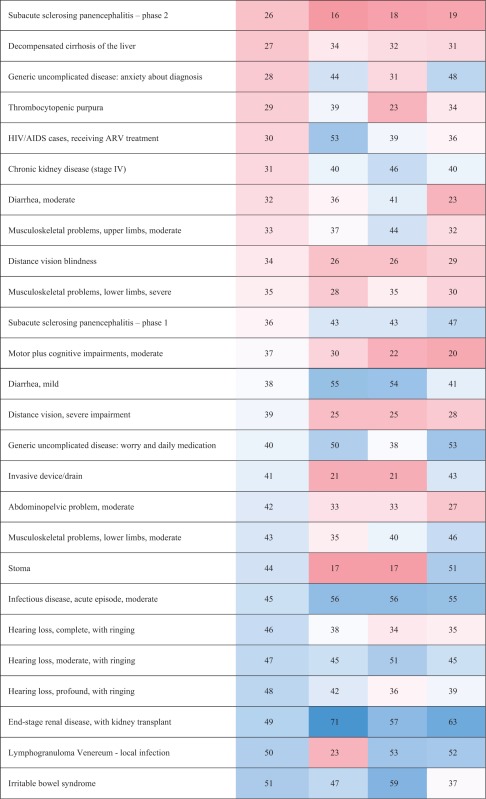
	(Continued)


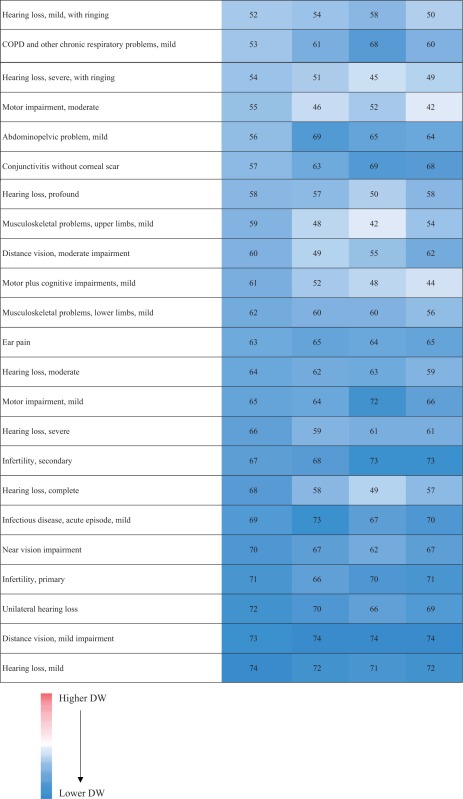

The median DW of all health states based on the responses of the Hungarian respondents was 0.118 (range: 0.007–0.597), 0.101 (range: 0.004–0.530) in Italian respondents, 0.108 (range: 0.004–0.624) in Dutch respondents and 0.111 (range: 0.005–0.717) in Swedish respondents. The range of DWs was higher in Swedish participants (0.712) and lower in Italian participants (0.526) but the shape of the DW distributions were right-skewed in all countries **(**Supplementary Appendix**)** and highly correlated **(***r*_s_ ≥ 0.99, *P* < 0.01), including in the range below 0.2, where most of the European DWs reside (*r*_s_ ≥ 0.97, *P* < 0.01).

The differences of DWs ranged from –0.135 (‘Thrombocytopenic purpura’) to 0.197 (‘Lymphogranuloma Venereum–local infection’) between Hungary and Italy, from –0.221 (‘AIDS cases, not receiving ARV treatment’) to 0.295 (‘Motor plus cognitive impairments, severe’) between Hungary and the Netherlands and from –0.165 (‘Musculoskeletal problems, generalized, severe’) to 0.250 (‘Stoma’) between Hungary and Sweden. The differences of DWs also ranked from –0.203 (‘End-stage renal disease, on dialysis’) to 0.226 (‘Motor plus cognitive impairments, severe’) between Italy and the Netherlands, from –0.187 (‘Intensive care unit admission’) to 0.241 (‘Stoma’) between Italy and Sweden and from –0.232 (‘Motor plus cognitive impairments, severe’) to 0.154 (‘Tuberculosis, not HIV infected’) between the Netherlands and Sweden. The biggest observed differences of DW were for ‘motor plus cognitive impairments, severe’ between Hungary and the Netherlands, between the Netherlands and Sweden and between Italy and the Netherlands (Hungary: 0.496; Italy: 0.426, The Netherlands: 0.200, Sweden: 0.432) and for ‘Stoma’, between Italy and Sweden and between Hungary and Sweden (Hungary: 0.301, Italy: 0.292, Sweden: 0.051). There were no differences in DW between Hungary and Netherlands for ‘Near Vision Impairment’, for ‘Distance vision, mild impairment’ and ‘Infertility, primary’ between Netherlands and Italy, for ‘Unilateral hearing loss’ between Netherlands and Sweden, for ‘Hearing loss, mild’, ‘Infectious disease, acute episode, mild’ and ‘Chronic kidney disease (stage IV)’ between Hungary and Sweden (Supplementary Appendix).

## Discussion

In this study, we aimed to assess the impact of country, sex, age, disease or injury experience status, educational and income levels on the evaluation of infectious disease health states.

First, we found a lower correlation of the probit coefficients between countries than between the other respondent characteristics. It means that we observed more variation of the health states valuation between countries than between the other respondent characteristics. Especially the correlation of the probit coefficient was lower between Hungary and the Netherlands than between the other pairs. This translated in some major differences, as for ‘motor plus cognitive impairments, severe’, that was ranked as the 25th worse health state in the Netherlands while instead fourth, seventh and ninth in Hungary, Italy and Sweden. We also observed that the model including country as covariate had the lowest AIC. This is in line with the results of the correlation analysis. The observed variations of health state valuation across country could reflect a combination of different demographics and/or different attitudes but also expressed that the included characteristics did not fully explain the between country differences.

We found a high correlation of the probit coefficients between educational levels, both across and within countries. This means that educational levels had no influence on health valuation. In 2008, Haagsma et al. also demonstrated no significant effects of educational level on DWs for injury consequences in Dutch population.^28^

In all countries, we observed a better correlation of the coefficients between 35–49 years and 50–65 years groups than between the other age groups. Dolan et al. also demonstrated that age group has an impact on health valuation.^15^^,^^16^

Within country we observed a lower correlation of the probit coefficients between income levels, especially between low and high income levels, except for the Netherlands where the lowest correlation was found between medium and high income levels.

Our results also showed that the probit coefficients did not vary widely across disease experience status and sex.

Although this study highlighted the effect of participants’ characteristic on health valuation using a sample of 30 660 answers, it also had some limitations.

First, we performed a web-based survey and did not include respondents aged 65 years or older. Elderly people are more often affected by some health states as hearing loss or distance vision loss, and may suffer of comorbidities.^29^ However, we did not demonstrate a relationship between DWs and disease experience status. Therefore this limitation may have had only a limited impact on the derived DWs.

Second, as in Salomon et al. in 2010, our study elicited DW by presenting brief health state descriptions in lay language and some aspects of health states were inevitably omitted in the interest of brevity. For example, people suffering from chronic irritable bowel syndrome need to undergo a restrictive diet which is not included in the health state description and can lead to a higher long-term disability.^30^ In addition, there may also have been differences between languages in the connotation of the words that were used in the health state descriptions. This could impact health state preferences of respondents and subsequently increase or decrease the value of the DW and impact the final ranking of the DWs.

Third, comparisons of DWs derived using a paired comparison approach across countries and participant characteristics are difficult and have to be interpreted with caution.^31^ Due to the nature of the paired comparison questions, the results hold only relative information. It is therefore not possible to compare the mean of all DWs across countries or between participants’ characteristics and for instance to conclude if one country has in average a higher or lower valuation of health. Instead, we explored different approaches for evaluating relative differences. First, we fitted different probit regression models to evaluate the interaction effects of country and participant characteristics. Comparison of models was only possible based on AIC values, which do not have an absolute interpretation and for which no null hypothesis distribution is available. AIC values thus do not allow to conclude on statistical significance of differences between models. Second, we used correlation coefficients to compare the rankings between different countries or participant characteristics. Because of the high number of observations, all correlation coefficients were significantly different from zero. Furthermore, the resulting correlation coefficients were all relatively high (>0.84). However, a definition of what could be considered sufficiently high depends on context and cannot be provided by statistical models. Although we observed a high agreement of the health states valuation for all sub-groups, we still observed variation of the ranking of DWs between some sub-groups. This has to be considered because that could have an impact on the final derived DWs and indirectly on burden of infectious diseases estimates and policy priorities.

Despite these limitations, we believe that this study brings an empirical basis for understanding the health state preferences related to infectious diseases of a diverse set of European respondents. We observed that health state evaluations are highly correlated across participants’ characteristics but that there exists some variation in health assessment across country and within country between income levels. This means that country and income levels may have an impact on health state valuation. These variations should be further explored in a systematic way, also in non-European countries. There is a need of future studies aiming to be more representative of study populations in future DW elicitation exercises. We also recommend future researches studying the effect of other characteristics of respondents on health assessment.

## Supplementary data

Supplementary data are available at *EURPUB* online.

## Supplementary Material

Supplementary Appendix
