# Supplementary material for: Disability weights for infectious diseases in four European countries: comparison between countries and across respondent characteristics
Source: Eur J Public Health. 2017 Sep 11;28(1):124–33. doi: 10.1093/eurpub/ckx090 (PMC5881674; doi:10.1093/eurpub/ckx090)

**APPENDIX**

1. ***Distribution of disability weights by country***


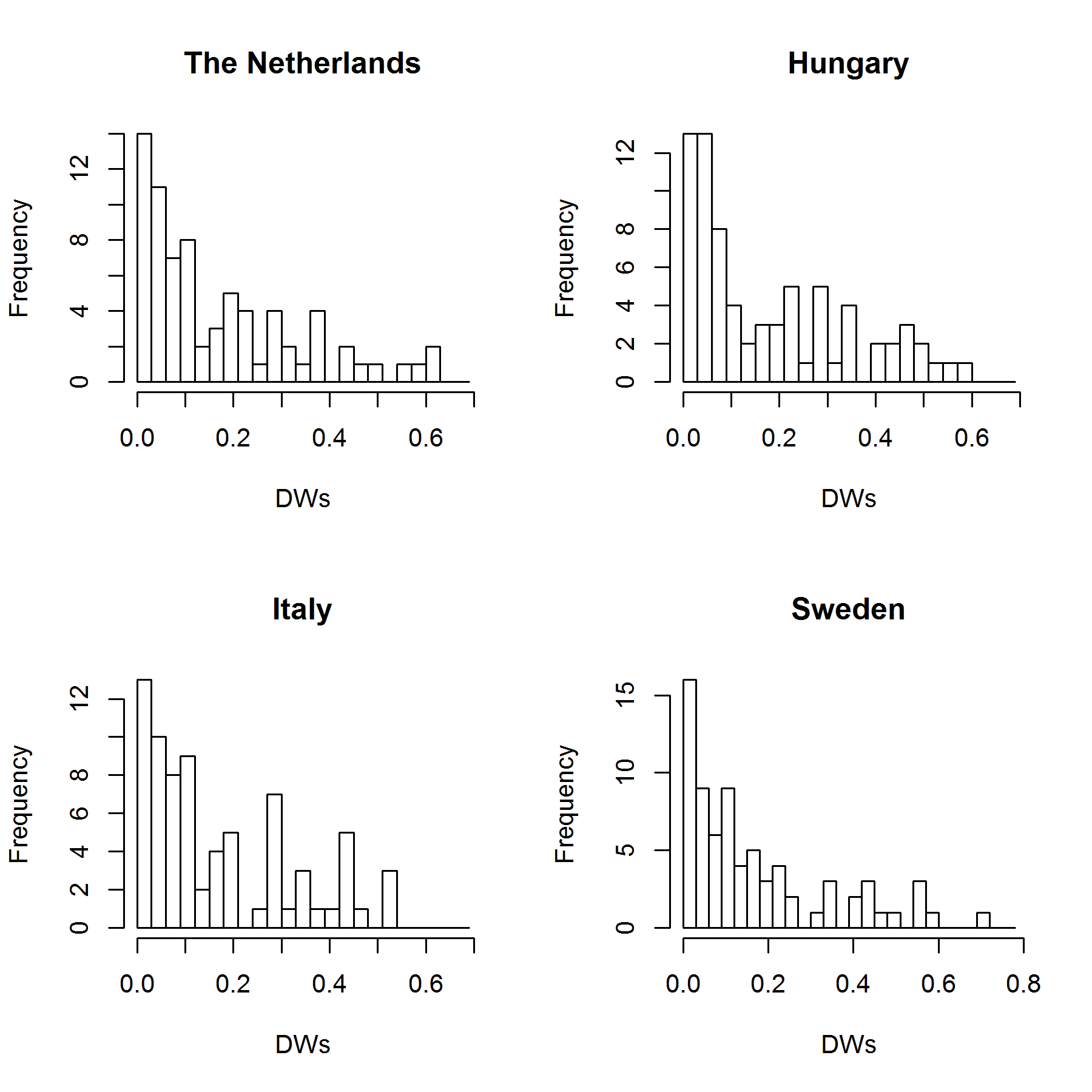


1. ***Distribution of the differences and 95% UI in elicited disability weights between countries***


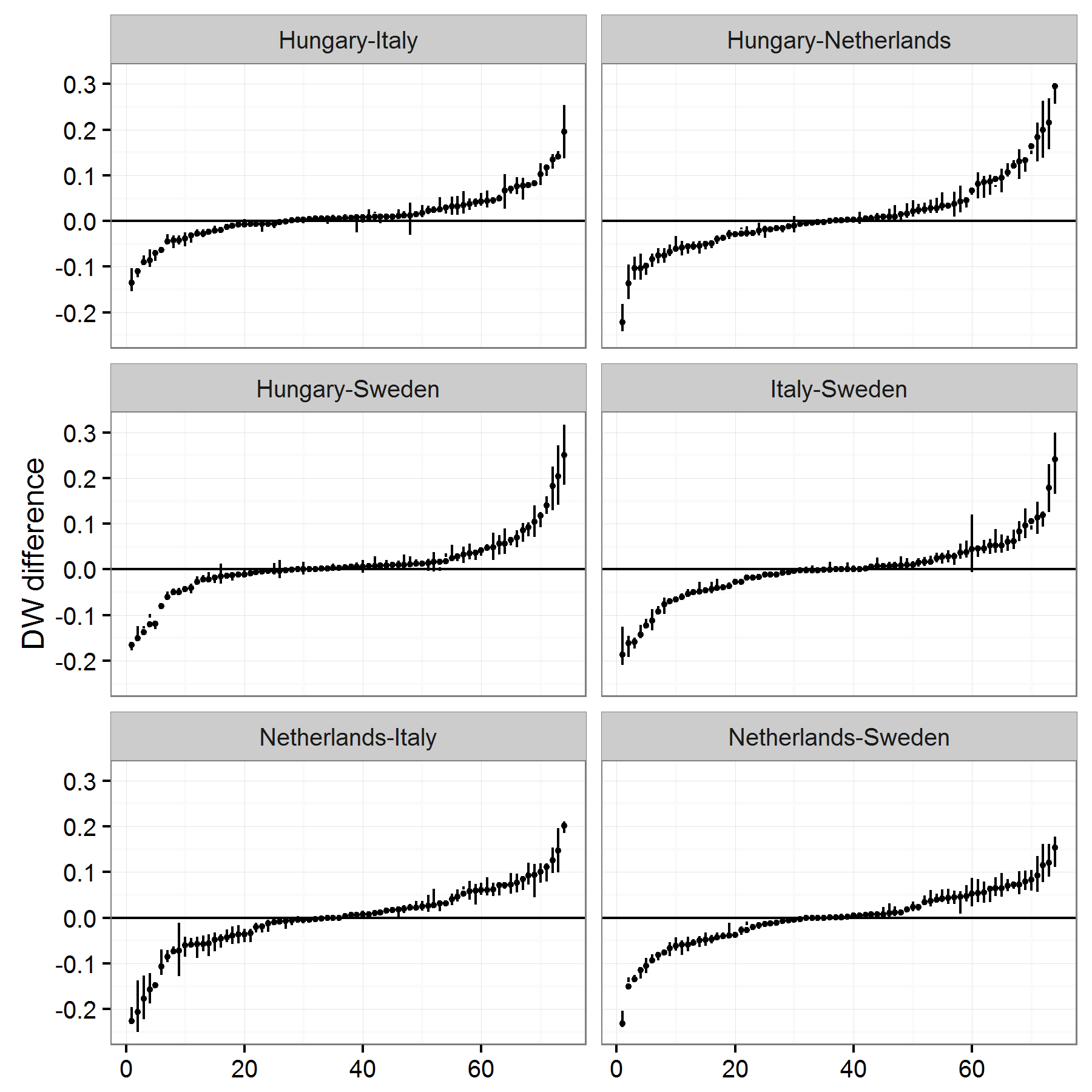

Supplement: Supplementary Appendix [file ejph-2016-10-om-0811-file004_ckx090.docx]
